# Mulberry fruit polysaccharides alleviate diethylnitrosamine/phenobarbital-induced hepatocarcinogenesis in vivo: the roles of cell apoptosis and inflammation

**DOI:** 10.1080/21655979.2021.1993716

**Published:** 2021-12-04

**Authors:** Shanshan Li, Yang Li, Hongjian Sun, Yang Jiang, Keming Pan, Yue Su, Nan Bu

**Affiliations:** a Jia Musi Hospital of Traditional Chinese Medicine, Jia Musi, Hei Longjiang, China; b Department of Gastroenterology, The First Affiliated Hospital of Harbin Medical University, Harbin, Hei Longjiang, China; c Department of Surgical Oncology, Jia Musi Central Hospital, Jia Musi, Hei Longjiang, China

**Keywords:** Mulberry, pro-apoptosis, anti-inflammatory, hepatocellular carcinoma, chemoprevention

## Abstract

Hepatocellular carcinoma (HCC) is one of the most common malignant tumors worldwide, and chemoprevention represents a feasible treatment to reduce the mortality of this carcinoma. Mulberry fruit polysaccharides (MFP) possess immunoregulatory and anti-inflammatory effects, which have been reported to alleviate liver damage evoked by CCl4 or alcohol in previous reports. However, its chemopreventive effect against liver carcinogenesis is insufficient. The present study was aimed to investigate the possible role of MFP as a pro-apoptosis, and anti-inflammatory agent to possess its chemoprevention property. Hepatocarcinogenesis was induced by diethylnitrosamine/phenobarbital (DEN/PB) for 14 weeks. The DEN/PB-administered rats were co-treated with different doses of MFP (50 or 100 mg/kg body weight) by oral gavage for 14 weeks. Basic hepatic function indexes (AST, ALT, ALP, GGT, total bilirubin, and albumin), and hepatic tumor biomarkers (AFP, CEA, and CA19.9), together with histological assessment were performed. Besides, the hepatic apoptosis markers (Bcl-2, Bax, caspase3, and caspase9), inflammation markers (IL-1β, TNF-α, and NF-κB), and mutT homologue gene 1 (MTH1) were examined. Oral gavage of MFP inhibited the elevations of hepatic function indexes and hepatic tumor biomarkers and alleviated pathological changes in hepatic tissue. In addition, the hepatic apoptosis markers, inflammation markers, and the mRNA level of MTH1 were abnormal in DEN/PB group, which were reversed by MFP treatment. In conclusion, MFP is an effective agent that provides chemoprevention against DEN/PB-evoked hepatocarcinogenesis via inhibition of inflammation and induction of apoptosis.

## Introduction

Liver cancer is one of the most common malignancies globally, and hepatocellular carcinoma (HCC) is the most fatal type of liver cancer that often induces as a consequence of chronic cirrhosis and liver disease [1]. Its incidence has risen steadily over the past few decades, and it has been the fastest-growing cause of cancer-related deaths in the USA [2,3]. The major risk factors for HCC are hepatitis B virus and hepatitis C virus infections, obesity, chronic alcohol use, food additives, aflatoxins, industrial and environmental toxic chemicals [4–6].

Although the detailed molecular mechanism of hepatocarcinogenesis remains unclear, accumulated evidence indicated that liver inflammation and dysregulation of apoptosis are important mechanisms involved in the pathogenesis of HCC [7,8]. Diethylnitrosamine (DEN) is a well-known hepatocarcinogenic agent present in agricultural chemicals, alcoholic beverages, cheddar cheese, cured and fried meals, and pharmaceutical products [9]. It has been reported that DEN could evoke liver cancer via induction of oxidative stress, cell apoptosis, and inflammation . Because of the carcinogenic properties of DEN, the administration of DEN in rodents is a well-established model of human HCC to investigate the pathogenetic changes underlying the progression of hepatocarcinogenesis [10].

At present, the transarterial, percutaneous, surgery, and drugs interventions are available for the treatment of HCC, however, the therapeutic outcome remains very poor [11]. Therefore, the efforts for discovering low toxic therapies targeting inflammation and apoptosis are necessary to manage HCC. Chemoprevention is an effective approach for cancer management, which is expected to prevent the initiation, promotion, and progression of carcinogenesis. In this regard, lots of natural products and their derived compounds have been investigated and proved efficacy for the prevention of hepatocarcinogenesis induced by DEN [12–15].

Mulberry (*Murus alba* L.) belongs to the *Moraceae* family, is widely distributed worldwide, especially in Africa, Asia, and Europe [16]. The fruit of Mulberry is consumed fresh or made into jam, fruit juice, or wine with multiple health benefits, such as antioxidant, antibiotic, hepatoprotective, and anti-inflammatory properties [17]. In China, it has also been used effectively as a folk medicine for its pharmacological activities, such as liver protection, anti-hypertension, fever reduction, and treatment of sore throat [18]. These beneficial effects of mulberry fruit are possibly attributable to active compounds, including anthocyanins, polysaccharides, flavonols, and polyphenols [18]. Mulberry fruit polysaccharides (MFP) are bioactive compounds that have been reported to exert hypolipidemic, hypoglycemic, anti-inflammatory, and hepatoprotective activities [16,19,20]. MFP protected LPS-stimulated macrophages from apoptotic cell death via modulating Bcl-2/Bak protein ratio [21]. However, there is little information on the beneficial effects of MFP against HCC.

According to these studies, we hypothesized MFP has a therapeutic effect in the treatment of HCC. To verify this, we investigated the potential therapeutic effects of MFP against DEN/PB-induced hepatocarcinogenesis in rats via targeting molecular parameters of apoptosis, and inflammation.

## Materials and methods

### Plant material and chemicals

Mulberry fruits were purchased from the Haozhou medicinal material market (Anhui, China), which were harvested in May of 2019 in the Xinjiang province. Diethylnitrosamine (DEN) and phenobarbital (PB) were purchased from Sigma-Aldrich (St. Louis, USA). All other chemical reagents used in the present study were of analytical grade.

### Preparation of MFP

MFP was prepared based on previous study [22]. About 3 kg of dried mulberry fruit was extracted with 80% ethanol for 24 h at room temperature to remove small lipophilic molecules and impurities. Subsequently, the extract was filtered and the resulting residue was freeze-dried for 48 h. Then, the residue was collected and extracted three times with 1 L water at 80°C (each time is 0.5 h). The extract was filtered again and concentrated in a vacuum. The condensate was precipitated with four volumes of absolute ethanol for 36 h at 4°C. The precipitate was obtained by centrifugation at 5000 *g* for 10 min. Finally, after the precipitate was washed with anhydrous ethanol and acetone, the crude MFP was obtained by freeze-drying.

## The chemical analysis of MFP

The weighted average molecular weight (Mw), numerically average molecular weight (Mn), and polydispersity (Mw/Mn) of MFP was measured by size exclusion chromatography equipped with a refractive index detector and Shodex-OHpak SB-804 HQ column (8.0 mm × 300 mm), eluted with 0.1% sodium chloride at a flow rate of 0.5 mL/min, and using the pullulan standard [23]. Carbohydrate content was measured by the phenol sulfuric acid method with D-glucose as the standard [24]. Uronic acid was determined based on the hydroxy diphenyl colorimetric method with GalA as the standard [25]. Sulfate content was measured by the barium chloride-gelatin method by using K_2_SO_4_ for the standard [26]. The protein content was measured according to the Bradford method with bovine serum albumin as standard [27].

### Experimental animals

Pathogen-free male Sprague-Dawley rats (weighing 120 ± 10 g) were purchased from the Animal Experiment Center of Hei Longjiang Province. All rats were housed in standard laboratory conditions (relative humidity 55 ± 5%, temperature 22 ± 2°C, and a 12–12 h light-dark cycle), and were allowed free access to standard chow and tap water *ad libitum*. All experimental procedures are following the instructions and guidelines for the care and use of experimental animals and are approved by the Animal Ethics Committee of Jia Musi Hospital of Traditional Chinese Medicine (Approval number: 20,200,096).

### Experimental design

**Figure 1.**
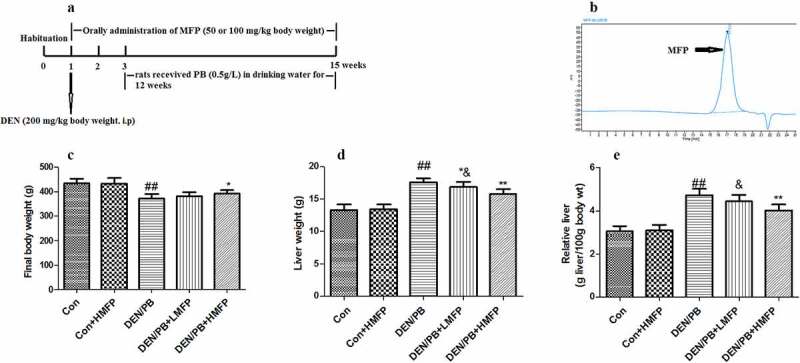
(a) Detailed experimental design. Physiological assays of rats with DEN/PB-induced HCC that were treated with Mulberry fruit polysaccharides (MFP). (b) The chromatograms of MFP. (c) Final body weight of rats with DEN/PB-induced HCC that was treated with MFP. (d) Liver weight of rats with DEN/PB-induced HCC that was treated with MFP. (e) Relative liver weight of rats. ^##^ Significant against Con group at *P* < 0.01. ** Significant against DEN/PB group at *P* < 0.01. * Significant against DEN/PB group at *P* < 0.05. ^&^ Significant against DEN/PB+HMFP group at *P* < 0.05

After acclimatization for 7 days, the pathogen-free rats were weighed and randomly assigned into five groups (each group consists of 12 rats). Control group (Con), rats received tap water by oral gavage. Con+HMFP group, rats received a high dose of MFP (HMFP) by oral gavage. DEN/PB group, rats received tap water by oral gavage and hepatocarcinogenesis was initiated by a single intraperitoneal injection with DEN (dissolved in 0.9% saline) at a dose of 200 mg/kg body weight [28]. Meanwhile, Con and Con+HMFP groups were similarly intraperitoneal injections with an equal volume of 0.9% saline. After two weeks of the recovery period, all DEN-initiated rats were received PB (the promoter PB was added daily into the tap water at the concentration of 0.5 g/L) for 12 successive weeks. In DEN/PB+LMFP and DEN/PB+HMFP groups, rats were administrated exactly as DEN/PB group and additionally orally administered a low dose of MFP (LMFP) at 50 mg/kg body weight or HMFP at 100 mg/kg body weight daily for 14 successive weeks. The choice of MFP dose and treatment time was based on previous literature [20,28,29]. The detailed timelines were shown in Figure 1a.

## Sample collections

At the end of the experiment, the rats were weighed and anesthetized by diethyl ether for 2–5 min. The blood sample was collected from the jugular vein and centrifuged at 4,000 *g* for 10 min at 4°C. The supernatant was collected and stored at −20°C for biochemical analysis. The right lobe of each liver was collected for all analyses, washed using ice saline, patted dry, and weighted. Then, liver tissues were homogenated with ice saline (1/9, m/v) using a homogenizer and centrifuged at 4,000 *g* for 10 min at 4°C, and the supernatant was collected and stored at −20 °C for biochemical analysis.

## Biochemical analysis of hepatic function and tumor biomarkers

Hepatic function markers, including aspartate transaminase (AST), alkaline transaminase (ALT), alkaline phosphatase (ALP), glutamyl transpeptidase (GGT), total bilirubin, and albumin levels were measured by the commercial kits (Nanjing Jiancheng Bioengineering Institute, Nanjing, China) according to the manufacturer’s instruction. The catalog numbers as follows: AST (C010-2-1), ALT (C009-2-1), ALP (A059-2-2), and GGT (C017-2-1).

Hepatic tumor biomarkers like serum levels of alpha-fetoprotein (AFP), carcinoembryonic antigen (CEA), and carbohydrate antigen 19.9 (CA19.9) were measured by ELISA kits (R&D Systems, Minneapolis, MN, USA) according to the manufacturer’s instruction. The catalog numbers as follows: AFP (ml063401), CEA (ml689701), and CA19.9 (ml058030).

## Determination of hepatic apoptosis markers

Hepatic caspase-3 and caspase-9 levels were measured using a kit purchased from Nanjing Jiancheng Bioengineering Institute (Nanjing, China) based on the manufacturer’s protocol. Briefly, 50 µl of sample solution and 50 µl of biotin-labeled antibody were incubated for 1 h at 37°C on a 96 well plate. Then, 80 µl of HRP-conjugated anti-rabbit Fab was added and incubated for 1 h at 37°C. The TMB substrate solution was added and incubated for 10 min at 37°C, and the reaction was stopped by the addition of 50 µl of sulfuric acid (0.1 M). Finally, the absorbance was measured at 450 nm. The catalog numbers are as follows: caspase-3 (G015-1-1) and caspase-9 (G018-1-2).

## Measurement of inflammation biomarkers

Hepatic interleukin-1beta (IL-1β), tumor necrosis factor-alpha (TNF-α), and nuclear factor-kappaB (NF-κB) levels were measured using a kit purchased from Nanjing Jiancheng Bioengineering Institute (Nanjing, China) based on the manufacturer’s protocol. The catalog numbers are as follows: IL-1β (H002), TNF-α (H052-1), and NF-κB (H202).

## Histopathological examination

At the end of the 15^th^ week, the rats were anesthetized by diethyl ether. And the liver tissues were removed quickly, excised and fixed with 4% paraformaldehyde overnight, and embedded in paraffin. Tissue samples were deparaffinized in xylene, dehydrated in alcohol. A thickness of 5 µm sections was cut and stained with hematoxylin and eosin. The pathological changes were observed using a light microscope (Olympus BX50, Japan) at a magnification of 100 × .

### Quantification of hepatic apoptosis and inflammation gene expression

A TRIzol reagent (Invitrogen, CA, USA) was used to separate total RNA from the liver tissue following the manufacturer’s protocols. The concentration and purity of RNA were measured by a UV Spectrophotometer (Thermo Scientific, USA). Then, 2 µg of RNA was transcribed to cDNA using PrimeScript^TM^ RT reagent kit (Thermo Scientific, USA). An SYBR advantage qPCR Master Mix kit (Thermo Scientific, USA) was used to perform RT-qPCR amplification following the manufacturer’s protocol. The sequences of the primers are shown in Table 1. GAPDH was used as an internal control.

**Table 1.** Sequences of primers used quantitative real-time PCR

Gene	Forward primer	Reverse primer
Bcl-2	5ʹ-ACTTCTCTCGTCGCTACCGTCGC-3’	5ʹ-AGAGCGATGTTGTCCACCAGGG-3’
Bax	5ʹ-CCAGGACGCATCCACCAAGAAG-3’	5ʹ-CCCAGTTGAAGTTGCCGTCTGC-3’
TNF-α	5ʹ-CCACGCTCTTCTGTCTACTG-3’	5ʹ-GCTACGGGCTTGTCACTC-3’
Caspase‑3	5ʹ-GGAGCAGTTTTGTGTGTGTGA-3’	5ʹ-TGTCTCAATACCGCAGTCCA-3’
Caspase‑9	5ʹ-TGGCATACACCCTGGACTC-3’	5ʹ-GCCGTGACCATTTTCTTAGC-3’
NF-κB	5ʹ-AGCACCAAGACCGAAGCAA-3’	5ʹ-TCTCCCGTA ACCGCGTAGTC-3’
IL-1β	5ʹ-GCCGATGGTCCCAATTACAT-3’	5ʹ-ACAAGACCTGCCGGAAGCT-3’
MTH1	5ʹ-GAGCGGCGGTGCAGAACCCAG-3’	5ʹ-AGAAGACATGCACGTCCATGAG-3’
GAPDH	5ʹ-CCCCCAATGTATCCGTTGTG-3’	5ʹ-TAGCCCAGGATGCCCTTTAGT-3’

### Immunohistochemistry for glutathione S-transferase P-form (GST-P), B-cell lymphoma-2 (Bcl-2), and NF-κB

The hepatic tissue samples were fixed in formalin (10%) and embedded in paraffin. The sections (5 µm thick) of the liver sample were dewaxed and hydrated progressively. Then, the sections were immersed in 0.1 mM sodium citrate buffer (pH 6) and placed in a microwave oven for 25 min to remove the antigen. After cool to room temperature, endogenous peroxidase activity was inhibited by exposure to H_2_O_2_ (0.3%). Then, the sections were incubated with GST-p (1:1000 dilutions, Medical and Biological laboratories Co., Tokyo), Bcl-2 (1:200 dilutions, Sigma Aldrich, MO. USA), and NF-κB (1:200 dilutions, Santa Cruz Biotechnology, USA) primary antibodies at 4°C overnight. Subsequently, the slides were incubated with a secondary antibody (horseradish peroxide-Polymer anti-Goat IgG) and visualized using a DAB Kit (Vector Laboratories, Burlingame, CA). Finally, the immunostaining was performed by light microscopy.

### Statistical analysis

Statistical comparison among different groups was performed using one-way ANOVA followed by Tukey’s multiple comparison test (Graphpad Software Inc., CA, USA). Data were reported as mean ± SD. Statistical significance was considered as *P* < 0.05.

## Results

A rat model of HCC was induced by DEN/PB, we investigated the potential therapeutic effect of MFP against HCC via evaluating the hepatic function indexes, hepatic tumor biomarkers, hepatic apoptosis markers, and inflammation markers. We demonstrated that the therapeutic effect of MFP inhibits tumorigenesis via inhibition of inflammation and induction of apoptosis.

### Chemical composition of MFP

As shown in Table 2, the Mw and polydispersity of MFP were 86.71 kDa and 1.15, respectively. Besides, the chemical composition of MFP contained carbohydrate (71.28 ± 1.21%), protein (2.63 ± 0.07%), uronic acid (20.31 ± 0.41%), and sulfate (4.01 ± 0.09%).

**Table 2.** The chemical analysis of polysaccharides from mulberry fruit

Sample	MFP
Mw (kDa)	86.71
Polydispersity (Mw/Mn)	1.15
Carbohydrate content (%)	71.28 ± 1.21
Protein	2.63 ± 0.07
Uronic acid	20.31 ± 0.41
Sulfate	4.01 ± 0.09

### Effect of MFP on hepatocarcinogenesis in DEN/PB-induced HCC

The relative liver weight, total number of nodules, tumor incidence, and mortality of rats were assessed to investigate the anti-cancer effects of MFP in DEN/PB-induced HCC (Figure 1 and Table 3). MFP decreased the relative liver weight (Figure 1d), the total number of nodules, tumor incidence, and mortality compared with those in the DEN/PB group. The results demonstrated that MFP exerted a potent anti-cancer effect in DEN/PB-induced HCC.

**Table 3.** Effect of MFP on mortality in DEN/PB-induced hepatocellular carcinoma

Group	Con	Con+HMFP	DEN/PB	DEN/PB+LMFP	DEN/PB+HMFP
Number of rats with nodules/number of rats	0/12	0/12	9/9	5/11	2/12
Total number of nodules	0	0	205	92**	36**
Average number of nodules/nodule-bearing liver (nodule multiplicity)	0	0	22.74 ± 1.83	18.80 ± 1.36**	17.06 ± 1.61**
Tumor incidence (%)	0	0	100	45.45**	16.67**
Number of death (n)	0	0	3	1	0
Number of rats (n)	12	12	12	12	12
Mortality (%)	0	0	25	8.33**	0**

** Significant against DEN/PB group at *P* < 0.01.

### Effect of MFP on hepatic function biomarkers in DEN/PB-induced HCC

**Figure 2.**
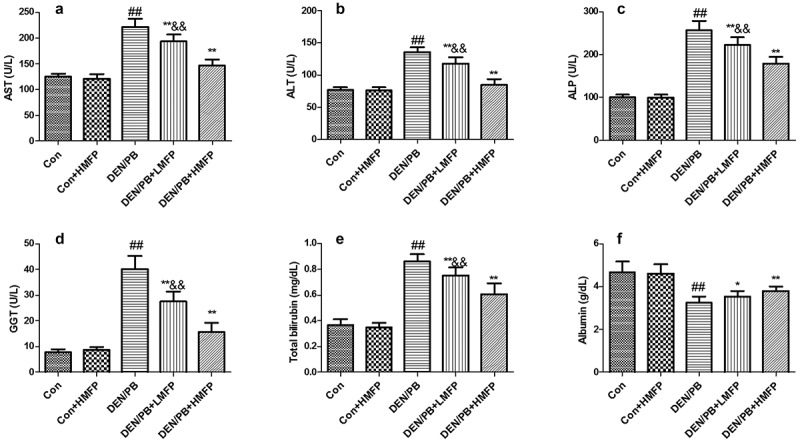
Effect of MFP on hepatic function biomarkers in DEN/PB-induced HCC. (a) The levels of serum aspartate aminotransferase (AST), (b) alanine aminotransferase (ALT), (c) alkaline phosphatase (ALP), (d) glutamyl transpeptidase (GGT), (e) total bilirubin, and (f) albumin in different treatment groups were measured. ^##^ Significant against Con group at *P* < 0.01. ** Significant against DEN/PB group at *P* < 0.01. * Significant against DEN/PB group at *P* < 0.05. ^&&^ Significant against DEN/PB+HMFP group at *P* < 0.01

Rats induced to develop liver damage exhibited a significant decrease in serum albumin and increases in serum AST (Figure 2a), ALT (Figure 2b), ALP (Figure 2c), GGT (Figure 2d), and total bilirubin (Figure 2e) levels and decrease in serum albumin (figure 2f). LMFP and HMFP groups showed an obvious increase in serum albumin and decrease in serum AST, ALT, ALP, GGT, and total bilirubin levels from DEN/PB group (*P* < 0.05 or *P* < 0.01).

### Effect of MFP on hepatic tumor markers in DEN/PB-induced HCC

**Figure 3.**
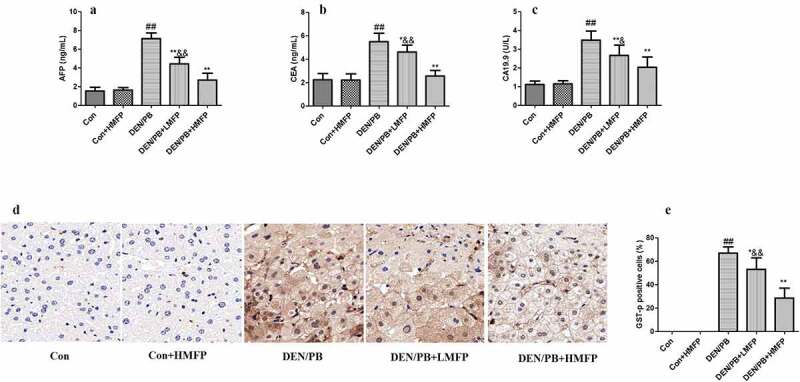
Effect of MFP on hepatic tumor markers in DEN/PB-induced HCC. (a) The levels of serum alpha-fetoprotein (AFP), (b) carcinoembryonic antigen (CEA), and (c) carbohydrate antigen 19.9 (CA19.9) in different treatment groups were measured. (d) Representative images of immunohistochemical staining with GST-p in different groups (scale bar = 100 μm). (e) The percentage of GST-p positive cells in different groups. ^##^ Significant against Con group at *P* < 0.01. ** Significant against DEN/PB group at *P* < 0.01. * Significant against DEN/PB group at *P* < 0.05. ^&^ Significant against DEN/PB+HMFP group at *P* < 0.05. ^&&^ Significant against DEN/PB+HMFP group at *P* < 0.01

Rats induced to develop liver cancer exhibited an obvious increase in serum AFP (Figure 3a), CEA (Figure 3b), and CA19.9 (Figure 3c) levels. LMFP and HMFP groups showed decreases in serum AFP, CEA, and CA19.9 levels from DEN/PB group (*P* < 0.05 or *P* < 0.01). Besides, as shown in Figure 3d-e, the percentage of GST-p positive cells has been increased in DEN/PB group. However, LMFP and HMFP groups showed decreases in the percentage of GST-p positive cells from DEN/PB group (*P* < 0.05 or *P* < 0.01).

### Effect of MFP on the hepatic apoptosis markers in DEN/PB-induced HCC

**Figure 4.**
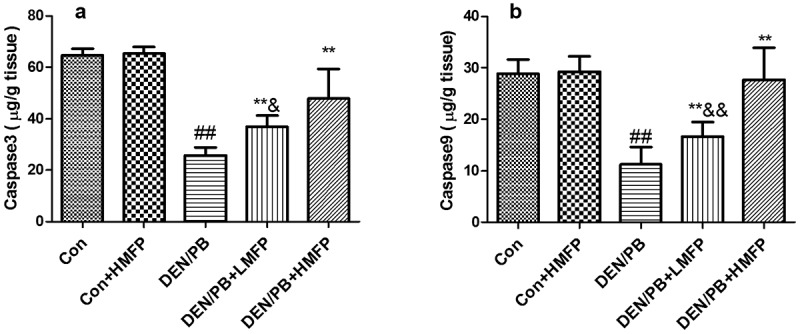
Effect of MFP on the hepatic apoptosis markers in DEN/PB-induced HCC. (a) The levels of hepatic caspase-3 and (b) caspase-9 in different treatment groups were measured. ^##^ Significant against Con group at *P* < 0.01. ** Significant against DEN/PB group at *P* < 0.01. ^&^ Significant against DEN/PB+HMFP group at *P* < 0.05. ^&&^ Significant against DEN/PB+HMFP group at *P* < 0.01

The normal hepatic apoptosis markers were changed in DEN/PB group, as revealed by the decreased hepatic caspase-3 (Figure 4a), and caspase-9 (Figure 4b) levels. LMFP and HMFP groups showed increases in hepatic caspase-3 and caspase-9 levels from DEN/PB group (*P* < 0.01).

### Effect of MFP on the hepatic inflammation markers in DEN/PB-induced HCC

**Figure 5.**
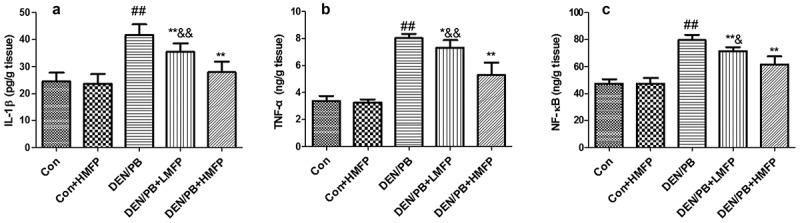
Effect of MFP on the hepatic inflammation markers in DEN/PB-induced HCC. (a) The levels of hepatic IL-1β, (b) TNF-α, and (c) NF-κB in different treatment groups were measured. ^##^ Significant against Con group at *P* < 0.01. ** Significant against DEN/PB group at *P* < 0.01. ^&^ Significant against DEN/PB+HMFP group at *P* < 0.05. ^&&^ Significant against DEN/PB+HMFP group at *P* < 0.01

The normal hepatic inflammation markers were altered in DEN/PB group, as revealed by the increased hepatic IL-1β (Figure 5a), TNF-α (Figure 5b), and NF-κB (Figure 5c) levels. LMFP and HMFP groups showed decreases in hepatic IL-1β, TNF-α, and NF-κB levels from DEN/PB group (*P* < 0.01).

### MFP boosted apoptosis of liver tissue in DEN/PB-induced HCC

**Figure 6.**
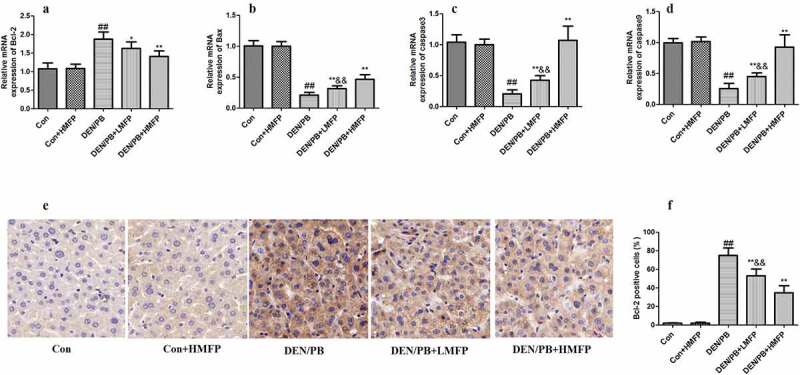
mRNA expression in rat’s hepatic tissue with DEN/PB-induced HCC. The level of Bcl-2 (a), Bax (b), caspase-3 (c), and caspase-9 (d) in different treatment groups. (e) Representative images of immunohistochemical staining with Bcl-2 in different groups (scale bar = 100 μm). (f) The percentage of Bcl-2 positive cells in different groups. ^##^ Significant against Con group at *P* < 0.01. ** Significant against DEN/PB group at *P* < 0.01. * Significant against DEN/PB group at *P* < 0.05. ^&&^ Significant against DEN/PB+HMFP group at *P* < 0.01

The mRNA expression level of Bcl-2 (Figure 6a) was up-regulated compared with that in the DEN/PB group (*P* < 0.01). LMFP and HMFP groups showed down-regulation in hepatic Bcl-2 levels from DEN/PB group (*P* < 0.01). In contrast, the mRNA expression level of BCL-2-associated X (Bax) (Figure 6b), caspase-3 (Figure 6c), and caspase-9 (Figure 6d) were down-regulated compared with that in the DEN/PB group (*P* < 0.01). LMFP and HMFP groups showed up-regulation in hepatic Bax, caspase-3, and caspase-9 levels from DEN/PB group (*P* < 0.01). Moreover, the percentage of Bcl-2 positive cells has been increased in DEN/PB group (Figure 6e-f). LMFP and HMFP groups showed decreases in the percentage of Bcl-2 positive cells from DEN/PB group (*P* < 0.01).

### MFP down-regulated hepatic mRNA of IL-1β, TNF-α, NF-κB, and MutT homolog 1 (MTH1) in DEN/PB-induced HCC

**Figure 7.**
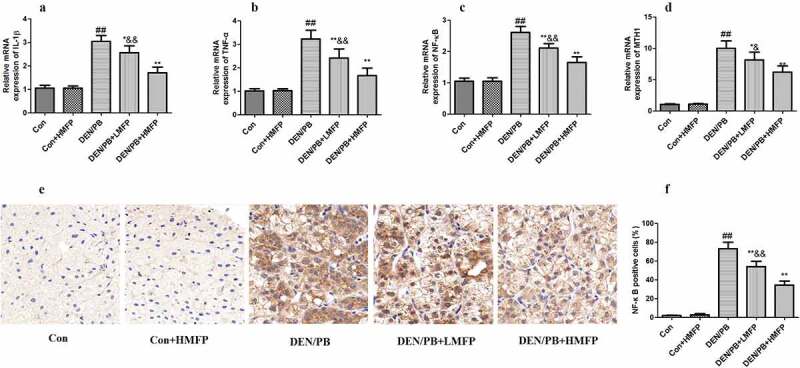
mRNA expression in rat’s hepatic tissue with DEN/PB-induced HCC. The level of IL-1β (a), TNF-α (b), NF-κB (c), and MTH1 (d) in different treatment groups. (e) Representative images of immunohistochemical staining with NF-κB in different groups (scale bar = 100 μm). (f) The percentage of NF-κB positive cells in different groups. ^##^ Significant against Con group at *P* < 0.01. ** Significant against DEN/PB group at *P* < 0.01. * Significant against DEN/PB group at *P* < 0.05. ^&^ Significant against DEN/PB+HMFP group at *P* < 0.05. ^&&^ Significant against DEN/PB+HMFP group at *P* < 0.01

The mRNA expression levels of IL-1β (Figure 7a), TNF-α (Figure 7b), NF-κB (Figure 7c), and MTH1 (Figure 7d) were up-regulated compared with these in the DEN/PB group (*P* < 0.01). LMFP and HMFP groups showed down-regulation in hepatic IL-1β, TNF-α, NF-κB, and MTH1 levels from DEN/PB group (*P* < 0.01). Additionally, the percentage of NF-κB positive cells has been increased in DEN/PB group (Figure 7e-f). LMFP and HMFP groups showed decreases in the percentage of NF-κB positive cells from DEN/PB group (*P* < 0.01).

### MFP alleviated DEN/PB-induced pathological changes

**Figure 8.**
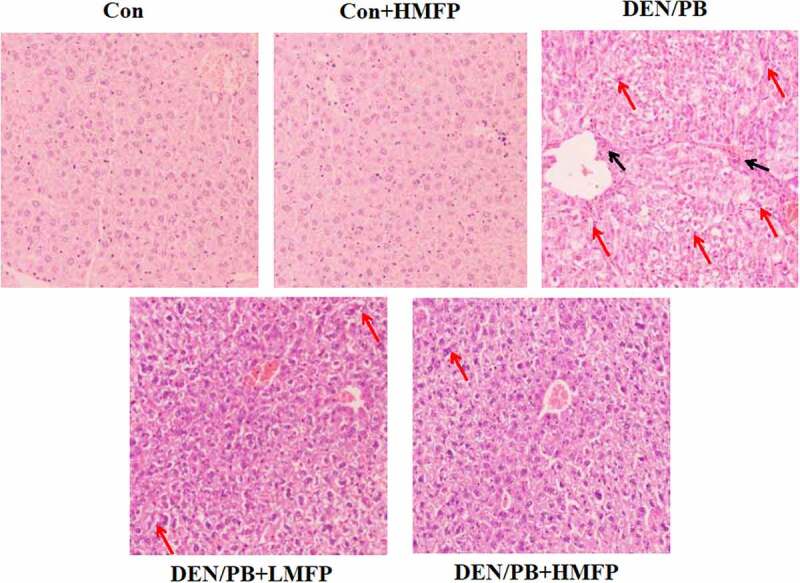
Liver tissues stained with H&E of all group rats. Histopathological assessment was performed under the microscope at 100 × . Black arrows indicate inflammation cell infiltration, red arrows indicate hepatocyte necrosis

H&E stained hepatic tissues from Con and Con+HMFP groups showed normal liver structure (Figure 8). Compared with the Con group, disorganized hepatocytes, severe hepatic inflammation cell infiltration, and hepatocyte necrosis were observed in livers of HCC-bearing rats. These pathological changes were improved to some degree in the DEN/PB+LMFP or DEN/PB+HMFP group.

## Discussion

HCC is one of the major causes of cancer-related death worldwide. Although there are different treatments for liver cancer, which including radiotherapy, chemotherapy, and liver transplantation, there are urgent needs for the development of alternative and effective therapies for liver cancer to avoid the high incidence and serious side effects of these traditional therapies [30,31].

In recent years, natural polysaccharides from bacteria, algae, fungi, and medical plants have attracted much attention due to their high medicinal value [32]. These bioactive polysaccharides could prevent the occurrence and development of all kinds of cancers, including colon cancer, lung cancer, and liver cancer [33]. In the present study, we extracted a bioactive polysaccharide from Mulberry (*Murus alba* L.). Intraperitoneal injection of DEN followed by promotion with PB for 14 consecutive weeks led to the development of hepatocarcinogenesis. Oral administration with MFP during this same period prevented the development of hepatocarcinogenesis.

DEN/PB could induce hepatic injury, which causing the destruction or deterioration of the cell membrane, ultimately resulting in the release of transaminases from the hepatic tissue. Liver enzymes, including AST, ALT, ALP, and GGT are the major indicator of hepatic disruption [34]. In our study, liver injury was induced by DEN/PB that was evidenced by the increased serum AST, ALT, ALP, and GGT levels, and the observed findings were in agreement with previous report [35]. In addition, the total bilirubin level increased, while serum albumin level declined in DEN/PB group. The oral treatment of DEN/PB-supplemented rats with MFP reversed the deteriorations in these hepatic function biomarkers. It is indicated that MFP helps liver cells regeneration, thus maintaining the integrity of the liver cell membrane, thereby reducing the leakage of these liver enzymes. In line with our findings, the hepatoprotective effect of MFP in the alcohol-induced liver damage model has been demonstrated [20].

It has been reported that the increased serum levels of AFP, CEA, and CA19.9 revealed the induction of HCC [12]. CEA and AFP are the major tumor biomarkers that previously demonstrated the HCC-bearing rats were found to have increased levels of these serum tumor markers [36]. In addition, AFP could regulate HCC cell growth and the suppression of AFP may be an effective treatment in HCC [37]. GST-P is overexpressed in the experimental hepatocarcinogenesis model and has been considered as a tumor marker of liver cancer [38]. In the present study, the oral treatment of DEN/PB-supplemented rats with MFP declined the increased levels of these serum tumor markers and the expression of GST-P, thus the MFP possesses potent anticancer activities against DEN/PB-induced HCC. Besides, histopathological examinations of liver tissue further confirmed the biochemical findings and showed the suppression of DEN/PB-evoked hepatocarcinogenesis.

Apoptosis plays an important role in cancer progression. Previous studies have indicated that tumor growth, survival, and proliferation are associated with the inhibition of extrinsic (death-receptor) and intrinsic (mitochondrial) pathways of apoptosis [39]. The mitochondrial apoptotic pathway was involved in the release of apoptotic cytokines and disruption of the mitochondrial membrane. Caspases are important mediators of apoptosis [40]. Activation of Caspase-9 triggers activation of Caspase-3, leading to biochemical and morphological changes related to apoptosis [41]. Besides, Bcl-2 families, including Bcl-2 and Bax, are considered as important mediators of cell apoptosis. Preclinical researches have indicated that drugs targeting members of the Bcl-2 family have clinical activity [42]. Thus, induction of apoptosis is the basic approach of cancer therapy [43]. The previous report has indicated that caspase-3 and Bcl-2 play an important role in Solanum nigrum polysaccharide-induced apoptosis [44]. In line with the current results, our findings indicated that MFP possessed an obvious proapoptotic effect, as evidenced by an increase in the expression of Bax, caspase-3, and caspase-9 and a decline in the expression of Bcl-2 compared with these in DEN/PB-treated rats. This result suggested that MFP prevented HCC progression in part via the induction of apoptosis.

Chronic hepatic inflammation could cause the injury of liver epithelial cells, which results in cell proliferation and cirrhotic. Thus, chronic hepatitis and the resulting cirrhotic microenvironment contribute to the development and progression of HCC [45]. The pro-inflammatory cytokines, including IL-1β, IL-6, TNF-α, and their downstream targets NF-κB promoted inflammation-associated HCC. NF-κB is a primary transcriptional regulator of genes involved in the apoptotic and inflammation processes of HCC progression. Increasing evidence has indicated that inhibition of NF-κB was recognized as effective cancer therapy and has attracted the attention of many cancer researchers [46–48]. In line with the previous reports [49,50], our findings demonstrated that HCC progression to be associated with increased hepatic inflammation with concomitant up-regulation of hepatic expression of IL-1β, TNF-α, and NF-κB. The oral treatment of DEN/PB-supplemented rats with MFP inhibited hepatic IL-1β, TNF-α, and NF-κB, thus MFP could improve liver inflammation and thus prevent the progression of HCC.

DNA injury is another important mechanism involved in the initiation and development of HCC, and MTH1 plays a vital role in the repair of oxidative DNA injury [51,52,53]. It has been reported that overexpression of MTH1 was observed in HCC tissue and downregulation of MTH1 may be a novel therapy for the treatment of HCC [14,53]. In line with these reports, we demonstrated that an up-regulation of hepatic expression of MTH1 was observed in HCC-bearing rats. The oral treatment of DEN/PB-supplemented rats with MFP suppressed hepatic MTH1 expression, thus MFP could induce the downregulation of MTH1 and thereby inhibit the tumorigenesis.

Here, there are several limitations to our experiments. Although the chemical analysis of MFP was performed in this study. However, the exact structure of MFP is not clear to us. In future experiments, it is necessary to conduct the structure characterization of MFP, such as HNMR, CNMR, and IR. Besides, although no serious side effects were observed. It is necessary to conduct safety experiments.

## Conclusion

Prevention of DEN/PB-evoked HCC by MFP was observed, as evidenced via the decrease the serum hepatic markers levels, as well as the hepatic tumor biomarkers levels. The well-recounted pro-apoptosis and anti-inflammatory effects of MFP were verified by the findings of our study, it was suggested to be involved in this anti-cancer effect of MFP. Taken together, our results implied that the activities of MFP inhibit the tumorigenesis via inhibition of inflammation and induction of apoptosis.

## Data Availability

The data that support the findings of this study are available from the corresponding author upon reasonable request.

## Highlights

1. MFP exerts chemoprevention effects against hepatocarcinogenesis.
2. MFP inhibits inflammation in DEN/PB-induced HCC.
3. MFP regulates apoptosis-related markers in DEN/PB-induced HCC.
4. MFP may be considered a novel agent against HCC.
